# Injured Achilles Tendons Treated with Adipose-Derived Stem Cells Transplantation and GDF-5

**DOI:** 10.3390/cells7090127

**Published:** 2018-08-31

**Authors:** Andrea Aparecida de Aro, Giane Daniela Carneiro, Luis Felipe R. Teodoro, Fernanda Cristina da Veiga, Danilo Lopes Ferrucci, Gustavo Ferreira Simões, Priscyla Waleska Simões, Lúcia Elvira Alvares, Alexandre Leite R. de Oliveira, Cristina Pontes Vicente, Caio Perez Gomes, João Bosco Pesquero, Marcelo Augusto M. Esquisatto, Benedicto de Campos Vidal, Edson Rosa Pimentel

**Affiliations:** 1 Department of Structural and Functional Biology, Institute of Biology, State University of Campinas–UNICAMP, Charles Darwin, s/n, CP 6109, 13083-970 Campinas, SP, Brazil; gianedc@gmail.com (G.D.C.); teo.luisfelipe@gmail.com (L.F.R.T.); daniloferrucci@yahoo.com.br (D.L.F.); gfsimoes2@gmail.com (G.F.S.); alroliv@unicamp.br (A.L.R.d.O.); crpvicente@gmail.com (C.P.V.); camposvi@unicamp.br (B.d.C.V.); pimentel@unicamp.br (E.R.P.); 2 Biomedical Sciences Graduate Program, Herminio Ometto University Center–UNIARARAS, 13607-339 Araras, SP, Brazil; marcelosquisatto@uniararas.br; 3 Department of Biochemistry and Tissue Biology, Institute of Biology, State University of Campinas–UNICAMP, Charles Darwin, s/n, CP 6109, 13083-970 Campinas, SP, Brazil; fernandaveiga6@gmail.com (F.C.d.V.); lealvare@unicamp.br (L.E.A.); 4 Engineering, Modeling and Applied Social Sciences Center (CECS), Biomedical Engineering Graduate Program (PPGEBM), Universidade Federal do ABC (UFABC), Alameda da Universidade s/n, 09606-045 São Bernardo do Campo, SP, Brazil; pritsimoes@gmail.com; 5 Department of Biophysics, Federal University of Sao Paulo–Unifesp, Pedro de Toledo, 699, 04039-032 Sao Paulo, SP, Brazil; caiopgomes@hotmail.com (C.P.G.); jbpesquero@unifesp.br (J.B.P.)

**Keywords:** repair, extracellular matrix, collagen, gait, biomechanics, gene expression

## Abstract

Tendon injuries represent a clinical challenge in regenerative medicine because their natural repair process is complex and inefficient. The high incidence of tendon injuries is frequently associated with sports practice, aging, tendinopathies, hypertension, diabetes mellitus, and the use of corticosteroids. The growing interest of scientists in using adipose-derived mesenchymal stem cells (ADMSC) in repair processes seems to be mostly due to their paracrine and immunomodulatory effects in stimulating specific cellular events. ADMSC activity can be influenced by GDF-5, which has been successfully used to drive tenogenic differentiation of ADMSC in vitro. Thus, we hypothesized that the application of ADMSC in isolation or in association with GDF-5 could improve Achilles tendon repair through the regulation of important remodeling genes expression. Lewis rats had tendons distributed in four groups: Transected (T), transected and treated with ADMSC (ASC) or GDF-5 (GDF5), or with both (ASC+GDF5). In the characterization of cells before application, ADMSC expressed the positive surface markers, CD90 (90%) and CD105 (95%), and the negative marker, CD45 (7%). ADMSC were also differentiated in chondrocytes, osteoblast, and adipocytes. On the 14th day after the tendon injury, GFP-ADMSC were observed in the transected region of tendons in the ASC and ASC+GDF5 groups, and exhibited and/or stimulated a similar genes expression profile when compared to the in vitro assay. ADMSC up-regulated *Lox*, *Dcn*, and *Tgfb1* genes expression in comparison to T and ASC+GDF5 groups, which contributed to a lower proteoglycans arrangement, and to a higher collagen fiber organization and tendon biomechanics in the ASC group. The application of ADMSC in association with GDF-5 down-regulated *Dcn*, *Gdf5*, *Lox*, *Tgfb1*, *Mmp2*, and *Timp2* genes expression, which contributed to a lower hydroxyproline concentration, lower collagen fiber organization, and to an improvement of the rats’ gait 24 h after the injury. In conclusion, although the literature describes the benefic effect of GDF-5 for the tendon healing process, our results show that its application, isolated or associated with ADMSC, cannot improve the repair process of partial transected tendons, indicating the higher effectiveness of the application of ADMSC in injured Achilles tendons. Our results show that the application of ADMSC in injured Achilles tendons was more effective in relation to its association with GDF-5.

## 1. Introduction

Tendon injuries are very common and their natural repair process is extremely slow, complex, and inefficient due to their intrinsic hypocellularity and hypovascularity, representing a clinical challenge to orthopedists mainly because these injuries often respond poorly to treatment [1]. As reviewed by Zabrzyński et al. [2], the occurrence of tendon injuries is associated with sports, aging, tendinophaties, hypothyroidism, hypertension, diabetes mellitus, arthropathies, corticosteroids, vitamin C deficiency, and others. Collagen fibers are the main component of the abundant extracellular matrix (ECM) of tendons, and with the proteoglycans (PG), the collagen fibers form a highly organized supramolecular structure, able to attend to the biomechanical demands on the tissue [3,4]. Particularly, small leucine-rich proteoglycans (SLRPs), such as decorin, fibromodulim, and biglycan, are the most abundant PG in tendons, with decorin representing 80% of the total proteoglycan content of the tissue [5]. SLRPs interact with the collagen fibrils, acting in the fibrillogenesis of collagen, and probably regulating the growth in diameter of these fibrils [6]. Besides the parallel bundles of predominantly type I collagen fibers and PG, non-collagenous glycoproteins, matrix metalloproteinases (MMP), growth factors, and cells also comprise elements of the tendons [5,7,8,9].

The specific mechanical tendon properties are directly related to the high organization of collagen bundles [10]. After injuries, the structural organization and composition of tendons are not completely restored, with a fibrous scar formation that can cause significant dysfunction and joint movement inability [11,12]. After the repair process, tendons become biomechanically weakened, making it more prone to re-rupture [9,13]. Clearly, new treatments are needed with the objective of improving tendon repair, considering that these injuries reach the population that is economically active, and that the use of cell therapy using mesenchymal stem cells (MSC) could be a good strategy.

Adult tissues are attractive MSC sources, which are characterized as undifferentiated cells, with mesodermal differentiation potential and self-renew and high proliferative capacities [14]. Adipose tissue is an alternative source of MSC that can be obtained by a less invasive method under local anesthesia, with little associated patient discomfort and in larger quantities as compared with bone marrow [15]. The effectiveness of adipose-derived mesenchymal stem cells (ADMSC) in regenerative medicine seems to be due to their paracrine effects that stimulate specific cellular events, like the growth factors and cytokines delivery [16], as well as their immunomodulatory effects to promote tendon regeneration [17]. Besides recruiting progenitor cells from some tissues, the ADMSC could also differentiate specific cells during tissue repair [18,19,20]. However, the literature is controversial about the effects of ADMSC transplantation during tendon repair. In some animal models, there was greater structural organization, biomechanical properties, density of collagen fibers, collagen types I and III genes expression, and growth factors synthesis as fibroblast growth factor (FGF), vascular endothelial growth factor (VEGF), and transforming growth factor (TGF-β) after ADMSC application in relation to untreated tendons [7,21,22,23]. Conversely, no differences were observed after this cellular therapy in the biomechanical parameters or collagen production [7,17,23].

It is widely accepted that the control of stem cell activity is influenced by several different environmental factors [24], including growth factors [1]. To drive tenogenic ADMSC in in vitro differentiation, insulin like growth factor (IGF)-1 or TGF-β in a co-culture with primary tenocytes and growth differentiation factor-5 (GDF-5) have been used successfully [19,20]. GDF-5 belongs to the TGF-β superfamily and it is known as cartilage-derived morphogenetic protein-1 (CDMP-1) and bone-derived morphogenetic protein-14 (BMP-14). Storm et al. [25] documented two other members of this superfamily, such as GDF-6 (BMP-13) and GDF-7 (BMP-12). GDF family members are also called bone-derived morphogenetic proteins (BMP) because they are found predominantly during the development of endochondral bones [26] and joint formation [27]. Wolfman et al. [28] demonstrated that the three members of BMP induce the formation of connective tissue rich in collagen I, with an important role in the process of tendon healing [29,30]. From this study, the BMP ceased to be considered as only chondrogenic and osteogenic factors, as suggested by the name, and were considered tendinogenic factors. Forslund and colleagues [30], who showed increased Achilles tendon resistance after rupture, reported the initial success of BMP-12, -13, and -14 in tendon healing. In a study of Chhabra et al. [31], mice deficient in the *GDF-5* gene had a poor healing process, with lesser structural organization and decreased biomechanical properties of tendons, evidencing the importance of this growth factor during tendon repair processes. Currently, cell therapy using the ADMSC associated with the exogenous application of growth factors represents a great potential in the process of tendon repair. Despite promising studies in animals, no treatment associated with the application of ADMSC in tendon injuries has been used in clinics due to the lack of knowledge on molecular aspects involving those therapies.

The objective of the present study was to test the hypothesis that the application of ADMSC in isolation or associated with GDF-5 could improve Achilles tendon repair. The use of GDF-5 was based on the literature that demonstrates its importance during tendon healing and the role of GDF-5 in modulating ADMSC tenogenic differentiation in vitro. Thus, the down- or up-regulation of remodeling genes expression in response to ADMSC and GDF-5 application were analyzed, and the involvement of those genes in the restoration of the structural, biomechanical, and functional properties of Achilles tendons after partial transection.

## 2. Materials and Methods

### 2.1. In Vitro Experiments

#### 2.1.1. Isolation of ADMSC and Cell Culture

The procedure was done according to Yang et al. [32] with some modifications. Adipose tissue was obtained from the inguinal region of 10 male Lewis rats between 90–120 days. Adipose tissue was cut and washed in Dulbecco’s modified phosphate buffered saline solution (DMPBS Flush without calcium and magnesium) containing 2% streptomycin/penicillin to remove contaminating blood cells. Then, 0.2% collagenase (Sigma-Aldrich^®^ Inc., Saint Louis, MO, USA) was added to degradation of the ECM and the solution was maintained at 37 °C under gentle stirring for 1 h to separate the stromal cells from primary adipocytes. Dissociated tissue was filtered using cell strainers (40 μm) and the inactivation of collagenase was then done by the addition of an equal volume of Dulbecco’s modified Eagle’s medium (DMEM) supplemented with 15% fetal bovine serum (FBS), followed by centrifugation at 1800 rpm for 10 min. The suspending portion containing lipid droplets was discarded and the pellet was resuspended in DMEM (containing 50 mg/L penicillin and 50 mg/L streptomycin) with 15% FBS, and transferred to 25 cm^2^ flasks for 48 hours. After confluence, cells were transferred to 75 cm^2^ flasks (1st passage). The medium was replaced after 48 h and then every 3 days. Cultures were maintained at 37 °C with 5% CO_2_ until the 5th passage (5P), always at up to 80% confluency.

#### 2.1.2. Flow Cytometry

ADMSC at 5P (*n* = 4) were trypsinized and centrifuged at 1800 rpm for 10 min, and counted using the Neubauer chamber. 1 × 10^6^ ADMSC were resuspended in 200 uL of DMPBS Flush with 2% BSA (bovine serum albumin). For the immunophenotypic panel, the following antibodies were used: CD90-APC, CD105-PE, and CD45-APC double conjugated (eBioscience^®^ Inc., San Diego, CA, USA) were diluted 1:200 and incubated for 40 min at room temperature. Subsequently, ADMSC were washed twice with 500 μL of DMPBS Flush and centrifuged at 2000 rpm for 7 min. The ADMSC were resuspended in DMPBS Flush with 2% BSA, followed by the flow cytometry analysis.

#### 2.1.3. In Vitro Differentiation Potential of ADMSC

Using different media for each type of differentiation, ADMSC (5P) were cultured (2 × 10^4^ cells) according to Yang et al. [32] with some modifications. Osteogenic differentiation (*n* = 3): DMEM supplemented with 10% FBS, 0.1 μM dexamethasone, 200 μM ascorbic acid, and 10 mM β-glycerol phosphate. Adipogenic differentiation (*n* = 3): DMEM supplemented with 10% FBS, 1 μmol/L dexamethasone, 50 μmol/L indomethacin, 0.5 mM 3-isobutyl-1-methyl-xanthine, and 10 μM insulin. Condrogenic differentiation (*n* = 3): DMEM supplemented with 10% FBS, acid free 15 mM HEPES, 6.25 μg/mL insulin, 10 ng/mL TGF-β1, and 50 nM AsAP. Cultures were maintained at 37 °C with 5% CO_2_, and the complete mediums were replaced twice a week. At the end of four weeks, cells were fixed with 4% paraformaldehyde for 20 min and stained with 2% Alizarin Red S (pH 4.1) during 5 min for calcium detection, with 0.025% Toluidine blue in McIlvaine buffer (0.03 M citric acid, 0.04 M sodium phosphate dibasic, pH 4.0) during 10 min for proteoglycans detection, and with 1% Sudan IV during 5 min to show lipid droplets. Samples were imaged on the Axiovert S100 (ZEISS) inverted microscope.

#### 2.1.4. Cell Viability

Flasks of 75 cm² with ADMSC in 5P (*n* = 3) were trypsinized and centrifuged at 1800 rpm for 10 min. The pellet obtained from each flasks was resuspended in 1 mL of DMPBS Flush. Then, an aliquot of 10 μL of each culture was stained with 0.4% Trypan Blue, and the ADMSC were placed for analysis in the Countess II FL (Life Technologies^®^ Inc., Carlsbad, CA, USA) equipment for cell concentration and viability measurements.

#### 2.1.5. Contrast by Differential Interference (DIC)

For the birefringence analyses of nuclei, ADMSC (5P) in culture (*n* = 3) were stained with 0.025% toluidine blue solution in 0.1 M McIlvaine phosphate buffer at pH 4.3 for 30 min, followed by analyses in the Olympus BX-51 (Olympus America, Center Vallery, PA, USA) polarizing microscope equipped with a Q-color 5 camera (Olympus America), and using the Image Pro-plus v.6.3 software for Windows™ (Media Cybernetics, Silver Spring, MD, USA) [4,33,34]. With the microscope and the software, it is possible to carry out analysis of DIC and the anisotropic properties, both individually and in combination [34]. DIC observations were performed after addition of the two condenser’s Wollaston prisms in the light path. With DIC-PLM, the optical path differences in samples can be detected through the sliding of another Wollaston prism that is positioned under the analyzer. The resulting colors in the sample are compared with the colors in a Michel-Lévy chart.

#### 2.1.6. Real Time-PCR Array for Analysis of ADMSC Gene Expression

For the total RNA extraction of the ADMSC (5P) using Trizol^®^ reagent, Invitrogen, Carlsbad, CA, USA, three different cultures were analyzed. A spectrophotometer (NanoDrop^®^ ND-1000, Thermo Fisher Scientific^®^, Waltham, MA, USA) was used to quantify the RNA in each sample by determining the absorbance ratio at 260 and 280 nm. 0.5 μg from the total extracted RNA of each sample was used for the synthesis of cDNA using the RT^2^ First Strand Kit (QIAGEN^®^, Hilden, Germany) and thermocycler Mastercycler Pro (Eppendorf^®^, Hamburg, Germany), also following the manufacturer’s instructions. The cDNA was frozen at −20 °C until tested. The RT-PCR array reaction was performed using the RT^2^ Profiler PCR Arrays (A format) kit in combination with the RT^2^ SYBR Green Mastermixes (QIAGEN^®^, Hilden, Germany) on the thermocycler apparatus 7300 (ABI Applied Biosystems^®^, Foster City, CA, USA), following the manufacturer’s instructions. For each culture sample, three types of reaction controls were used: 1. Positive PCR control; 2. Reverse transcriptase control; and 3. Control for contamination of rat genomic DNA. The *Glyceraldehyde-3-phosphate dehydrogenase* (*Gapdh*, NM_017008) was used as an endogenous control for each sample. The following genes were analyzed (QIAGEN^®^): *Scleraxis* (*Scx*, NM_001130508); *Tenomodulin* (*Tnmd*, NM_022290); *Tumor necrosis factor* (*TNF superfamily*, *member 2*) (*Tnf*, NM_012675); *Interleukin 1 beta* (*II1b*, NM_031512); *Transforming growth factor*, *beta 1* (*Tgfb1*, NM_021578); *Matrix metallopeptidase 2* (*Mmp2*, NM_031054); *Matrix metallopeptidase 9* (*Mmp9*, NM_031055); *Matrix metallopeptidase 8* (*Mmp8*, NM_022221); *TIMP metallopeptidase inhibitor 2* (*Timp2*, NM_021989); *Decorin* (*Dcn*, NM_024129); *Lysyl oxidase* (*Lox*, NM_017061); and *Growth differentiation factor 5* (*Gdf5*, XM_001066344). Reactions were made in a single cDNA pipetting for each gene, including the endogenous control. ∆CT values were obtained by the difference between the CT values of the target genes and the *Gapdh* gene. The 2^−∆CT^ method [35] was used to calculate the gene expression for each target gene.

### 2.2. In Vivo Experiments

#### 2.2.1. Experimental Groups

A total of 110 male Lewis rats (120-day-old), with free access to food and water, were divided into 5 experimental groups (22 animals for each group): Normal (N): Rats with tendons without transection; Transected (T): Rats with partially transected tendons and treated with topical application of DMPBS Flush in the transected region; Mesenchymal stem cells derived from adipose tissue (ASC): Rats with transected tendons with subsequent transplant of ADMSC (3.7 × 10^5^ cells) in the transected region; GDF-5 (GDF5): Rats with transected tendons and treated with topical application of DMPBS Flush in the transected region + GDF5 application (500 ng) 24 h after partial transection; and with ADMSC and GDF-5 (ASC+GDF5): Rats with transected tendons and treated with subsequent transplant of ADMSC (3.7 × 10^5^ cells) in the transected region + GDF5 application (500 ng) 24 h after partial transection. Animals were euthanized on the 14th day after transection by an overdose of anesthetic (Ketamine and Xylazine). Animals of group N were euthanized at 134 days and the tendons without transection were collected for analysis.

#### 2.2.2. Partial Transection of the Calcaneal Tendon and Application of ADMSC and GDF-5

The animals were anesthetized with intraperitoneal injection of Ketamine (90 mg/Kg) and Xylazine (12 mg/Kg), and the right lower paws submitted to antisepsis and trichotomy. For the exposure of the calcaneus tendon, a longitudinal incision was made in the animal’s skin, followed by a transverse partial transection performed in the proximal tendon region located at a distance of 4 mm from its insertion in the calcaneus bone [36,37]. Approximately 3.7 × 10^5^ ADMSC (5P) were resuspended in 15 μL of DMPBS Flush and transplanted in the transected region of tendons in the ASC group using a pipette. 15 μL of DMPBS Flush was applied in tendons of the T group, and the GDF5 group tendons received an application of 15 μL of DMPBS Flush + 500 ng of GDF-5 24 h after the tendon transection. All applications were made in the region of the tendon where the partial transection was performed. Then, the skin was sutured with nylon thread (Shalon 5-0) and a needle (1.5 cm). All surgical and experimental protocols were approved by the Institutional Committee for Ethics in Animal Research of the State University of Campinas-UNICAMP-Brazil (Protocol nº 2905-1).

#### 2.2.3. Preparation of Sections in Freezing

Tendons were placed in Tissue-Tek^®^, frozen, and cut in cryostat (serial longitudinal cuts of 7 μm thickness). The sections were fixed using a 4% formaldehyde solution in Millonig buffer (0.13 M sodium phosphate and 0.1 M sodium hydroxide, pH 7.4) for 20 min, followed by birefringence and linear dichroism analysis.

#### 2.2.4. DAPI Staining

Immediately after fixation, the sections (*n* = 4) were incubated with DAPI (4′,6-Diamidino-2-phenylindole dihydrochloride) (0.1 mg/mL in methanol) for 5 min at 37 °C. The sections were analyzed by fluorescence microscope (Olympus BX60) and the images captured by the QCapture 4.0 program.

#### 2.2.5. Polarization Microscopy: Birefringence Measurements

After fixation, image analyses of the tendons (*n* = 4) were evaluated to detect differences in morphology based on the aggregation and organization of the collagen bundles, which reflect the variation of birefringence intensity. Birefringence properties were studied using an Olympus BX53 polarizing microscope and an image analyzer (Life Science Imaging Software, Version 510_UMA_cellSens16_Han_en_00). Because the birefringence appears visually as brilliance, this phenomenon was measured with an image analyzer and expressed as gray average (GA) values in pixels (8 bits = 1 pixel). The larger tendon axis was positioned at 45° to the crossed analyzer and polarizer. As collagen bundles exhibit two types of birefringence, intrinsic birefringence (Bi) and form or textural birefringence (Bf) [38], the total birefringence (the sum of Bi and Bf) was used in this study. Measurements of the tendons in each experimental group were made after immersing the sections in water, a condition in which total birefringence is highly detectable [4,33,34,38]. The number of measurements of GA was represented as the median and they were chosen at random in 16 sections from four tendons of each group.

#### 2.2.6. Linear Dichroism Measurements

Linear dichroism measurements (*n* = 4) were obtained from toluidine blue-stained sections. Linear dichroism measurements have shown that GAG chains present in collagen fiber PGs are linearly distributed and predominantly parallel to the longest fiber axis [3,39,40]. In this case, linear dichroism is an extrinsic phenomenon, resulting from the arrangement of toluidine blue molecules that are electrostatically bound to the anionic link sites of the oriented substrate. The dichroic ratio (DR=dוו/d_┴_) was determined by the toluidine blue absorbance in the parallel (dוו) and perpendicular (d_┴_) positions of the tendon’s longest axis, with regard to the polarized light plane (PLP) [39,40]. Linear dichroism was measured using an Olympus BX53 polarizing microscope (Objective: Olympus UPlanFL N 40×; Camera: Olympus Q-color 5; Polarizer: Olympus U-POT) and an image analyzer (Life Science Imaging Software, Version 510_UMA_-cellSens16_Han_en_00). The number of measurements (~100) of GA was represented as the median and they were chosen at random in 16 sections from four tendons of each group.

#### 2.2.7. Real Time-PCR Array

The collected tendons (*n* = 4) were placed in stabilizing solution (RNA-later, QIAGEN^®^ Hilden, Germany) and maintained at −20 °C. For the total RNA extraction, the tendons were sprayed using liquid nitrogen and then homogenized in a tube containing 5 stainless steel balls (2.3 mm diameter, Biospec) by being shaken in a TissueLyser LT instrument (QIAGEN^®^ Hilden, Germany), with 2 repetitions (60 s) intercaleted with ice cooling (2 min) between each shaking step (5). Total RNA was isolated from each sample using the RNeasy^®^ Fibrous Tissue Mini Kit (QIAGEN^®^ Hilden, Germany), following the manufacturer’s instructions. A spectrophotometer (NanoDrop^®^ ND-1000, Thermo Fisher Scientific^®^, Waltham, Massachusetts, USA) was used to quantify the RNA in each sample by determining the absorbance ratio at 260 and 280 nm. 0.5 μg from the total extracted RNA of each sample was used for the synthesis of cDNA using the RT^2^ First Strand Kit (QIAGEN^®^ Hilden, Germany) and thermocycler Mastercycler Pro (Eppendorf^®^ Hamburg, Germany), also following the manufacturer’s instructions. The cDNA was frozen at −20 °C until tested. The RT-PCR array reaction was performed using the RT^2^ Profiler PCR Arrays (A format) kit in combination with the RT^2^ SYBR Green Mastermixes (QIAGEN^®^ Hilden, Germany) on the thermocycler apparatus 7300 (ABI Applied Biosystems^®^, Foster City, CA, USA), following the manufacturer’s instructions. For each animal sample, three types of reaction controls were used: 1. Positive PCR control; 2. Reverse transcriptase control; and 3. Control for contamination of rat genomic DNA. The *Glyceraldehyde-3-phosphate dehydrogenase* (*Gapdh*, NM_017008) was used as an endogenous control for each sample. The following genes were analyzed (QIAGEN^®^ Hilden, Germany): *Scleraxis* (*Scx*, NM_001130508); *Tenomodulin* (*Tnmd*, NM_022290); *Tumor necrosis factor* (*TNF superfamily*, *member 2*) (*Tnf*, NM_012675); *Interleukin 1 beta* (*II1b*, NM_031512); *Transforming growth factor*, *beta 1* (*Tgfb1*, NM_021578); *Matrix metallopeptidase 2* (*Mmp2*, NM_031054); *Matrix metallopeptidase 9* (*Mmp9*, NM_031055); *Matrix metallopeptidase 8* (*Mmp8*, NM_022221); *TIMP metallopeptidase inhibitor 2* (*Timp2*, NM_021989); *Decorin* (*Dcn*, NM_024129); *Lysyl oxidase* (*Lox*, NM_017061); and *Growth differentiation factor 5* (*Gdf5*, XM_001066344). Reactions were made in a single cDNA pipette for each gene, including the endogenous control. ∆CT values were obtained by the difference between the CT values of the target genes and the *Gapdh* gene. These values were normalized by subtracting the ∆CT value of the calibrator sample (T group) to obtain ∆∆CT values. A 2^−∆∆CT^ method was used to calculate the relative expression level (fold change) for each target gene. Results were represented as the relative gene expression in comparison to the calibrator sample that is equal to 1.

#### 2.2.8. Dosage of Hydroxyproline

Hydroxyproline was used as an indicator of the amount of total collagen in the tendons of the different groups (*n* = 5) used previously in the biomechanical assay. The tendons were cut and immersed in acetone for 48 h, followed by a solution containing chloroform:ethanol (2:1) also for 48 h. After dehydration, the samples were placed for drying in the oven at 37 °C. Samples were weighed and hydrolyzed in 6N HCl (1 mL/10 mg of tissue) for 4 h at 130 °C according to Stegemann and Stalder [41] with some modifications, and neutralized with 6N NaOH. The absorbance of the samples was measured at 550 nm using a microplate reader (Expert Plus, Asys^®^, Holliston, MA, USA).

#### 2.2.9. Evaluation of the Max Contact Intensity of the Rat Paw after Partial Transection

The CatWalk system (Noldus Inc., Wageningen, The Netherlands) was used to analyze the gait recovery of the animals (*n* = 5). In this protocol, the rats crossed a walkway (100 cm length, 5 cm width, and 0.6 cm thickness) with a glass floor illuminated from the long edge in a dark room. Data acquisition was performed with a high-speed camera (Pulnix TM-765E CCD), and the paw prints were automatically classified by the software. The paw prints were obtained during the 3 days before the partial transection of the tendons to assess the normal standard gait of the animals, and they were collected again after the lesions. Post-operative data were assessed on the 1st, 3rd, 5th, 7th, 9th, 11th, and 13th days following surgical lesion. The parameters used herein were “Max Contact Intensity”, corresponding to the pressure exerted by the paw on the glass floor during gait. The intensity of magnification can vary from 0 to 255 pixels.

#### 2.2.10. Biomechanical Parameters

Tendons from experimental groups (*n* = 5) were collected and stored at −20 °C until tested. Before the biomechanical test, the tendons were thawed and measured with pachymeter, considering their length, width, and thickness. For the biomechanical assay, the tendons were maintained in PBS to prevent their fibers from drying out. Then, the tendons were fixed to metal claws by the myotendinous junction and by the osteotendinous junction for correct alignment in the equipment (Texturometer, MTS model TESTSTAR II). In each biomechanical assay, tendons were subjected to a gradual increase of load at a displacement velocity of 1 mm/s by using a load 0.05 N until the tendon ruptured. Biomechanical parameters were analyzed according to Biancalana et al. [42] and Tomiosso et al. [9], such as maximum force (N) and maximum displacement (mm), which were used to calculate the maximum stress (Mpa) and maximum strain (L) of tendons from the experimental groups. The cross-sectional area of the calcaneus tendon was calculated by assuming an elliptical approximation (A = πWd/4) using measurements of width (W) and thickness (d) values from the same Sparrow et al. [43]. The maximum stress value (MPa) was estimated by the ratio between the maximum load (N) and the cross-sectional area (mm^2^). The maximum deformation (L) was calculated through (L = L_f_ − L_i_/L_i_), where (L_f_) is the value of the final length before rupture, and (L_i_) is the initial tendon length value. Stress-strain curves were constructed using the mean of each mechanical property obtained for each group.

#### 2.2.11. Statistical Analysis

All results were presented as the mean and standard deviation for the values with a normal distribution (or interquartile range and median for the values that did not adhere to the Gaussian distribution). For the data with normal distribution, the analysis of variance (ANOVA) was used, followed by the Tukey post-hoc test for intra-group analysis (in the case of statistical significance), or the Student’s *t* Test preceded by the Levene Test. For data that did not adhere to the Gaussian distribution, the non-parametric test of the Kruskal-Wallis test followed by the post-hoc test of Dunn for intra-group analysis (in the case of statistical significance) was used or the U Test of Mann-Whitney. Statistical analysis was performed in the software Statistical Package for Social Sciences (SPSS) version 22.0 and for all the aforementioned tests the significance level α = 0.05 and power of the test of 95% were considered.

## 3. Results

### 3.1. In Vitro

#### 3.1.1. In Vitro Differentiation Potential of ADMSC and Flow Cytometry

**Figure 1:**
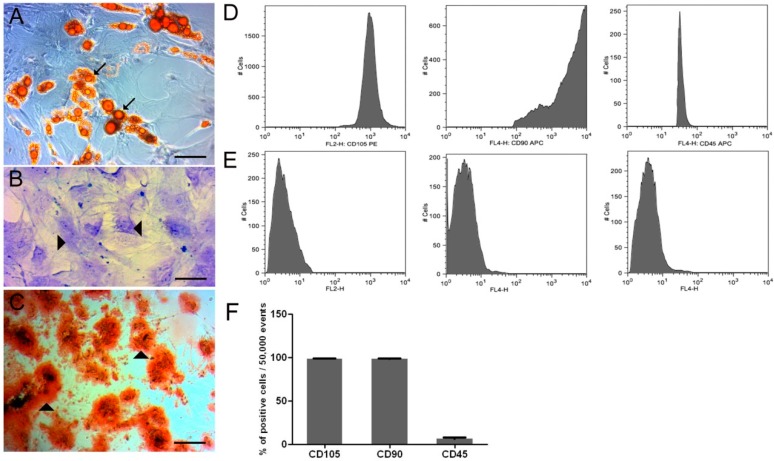
In vitro adipogenic (**A**), condrogenic (**B**), and osteogenic (**C**) differentiation of adipose-derived mesenchymal stem cells (ADMSC) in 5P: Observe intracellular lipid droplets stained with Sudan IV (→), proteoglycans stained with toluidine blue (▶), and extracellular calcium stained with alizarin red S (▶). Bars = A and C: 200 μm; B: 100 μm. (**D**) Histograms demonstrate the x-axis fluorescence scale considered positive when the cell peak is above 10^1^ (CD45) or 10^2^ (CD105 and CD90). (**E**) Control for –PE and –APC (with very low fluorescence), corresponding to non-marked cells. (**F**) Flow cytometry of ADMSC for CD105, CD90, and CD45 surface markers.

In vitro ADMSC staining with Toluidine Blue, Alizarin Red S, and Sudan IV showed differentiation of ADMSC at the 5P in chondrocytes, osteoblasts, and adipocytes, respectively (Figure 1A–C). Flow cytometric analysis (Figure 1D,E) showed the presence of CD90 (90%) and CD105 (95%) positive surface markers, and the negative marker, CD45 (75%).

#### 3.1.2. Fluorescence, Birefringence, and Contrast by Differential Interference (DIC)

**Figure 2:**
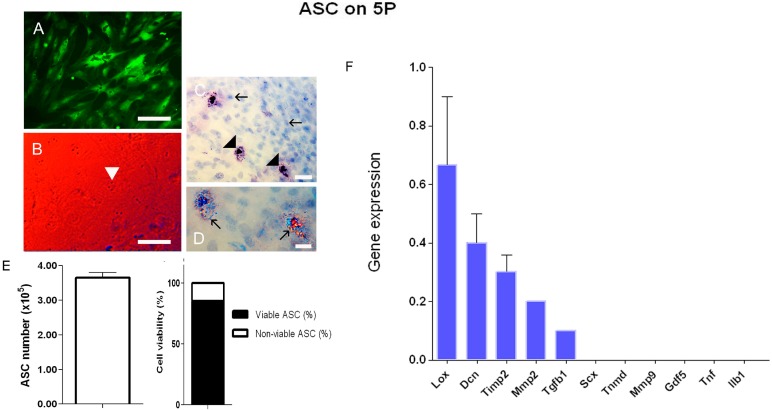
Morphology of ADMSC on 5P: (**A**) ADMSC-GFP: Fibroblast-like morphology, with a fusiform shape. (**B**) Contrast by Differential Interference (DIC): The red to blue band showing the interference effect of the nucleus due to higher concentrations of material. Nucleoli presence seen in the initial of the blue band (▶). (**C**) ADMSC stained with AT (pH 4.3): Note the presence of nuclei stained in blue (→), and the presence of granules of various sizes highly stained in blue in some regions (▶) due to the disponibility of phosphate groups. (**D**) Polarization microscopy: In the detailed image, note the abnormal interference colors due to abnormal dispersion of birefringence because of the differences in the high packing of DNA. Bars = 50 μm (**A**), 70 μm (**B**,**C**), and 35 μm (**D**). (**E**) Number of ADMSC used for application in tendons, presenting about 80% viability after tripsinization. (**F**) RT-PCR array of ADMSC on 5P in vitro showing the expression profile of the genes *Lox*, *Dcn*, *Timp2*, *Mmp2*, and *Tgfb1*. No expression was observed for *Scx*, *Tnmd*, *Mmp9*, *Gdf5*, *Tnf*, and *Ilb1* genes.

Analysis using fluorescence microscopy showed ADMSC-GFP on 5P with fusiform fibroblast-like morphology (Figure 2A). When DIC was used, nucleoli were observed (Figure 2B). On AT (pH 4.3) staining, most of the nuclei were stained in blue, with the presence of granules of various sizes in some regions being highly stained in blue (Figure 2C). Under polarizing microscopy, the granules exhibited abnormal interference colors due to differences in the high packing of DNA (Figure 2D).

#### 3.1.3. Cell Viability

Figure 1E showed a mean of 3.7 × 10^5^ ADMSC used for tendons’ application of the ADMSC and ASC+GDF5 groups, with a mean of 80% of viable ADMSC (Figure 2E).

#### 3.1.4. Real-Time PCR Array

In the RT-PCR array analysis of ADMSC on 5P, the expression profile of the genes, *Lox*, *Dcn*, *Timp2*, *Mmp2*, and *Tgfb1* was observed. No expression was observed for the *Scx*, *Tnmd*, *Mmp9*, *Gdf5*, *Tnf*, and *Ilb1* genes. The expression of the *Mmp8* gene was not represented because only one culture expressed it (Figure 2F).

### 3.2. In Vivo

#### 3.2.1. Immunofluorescence

**Figure 3:**
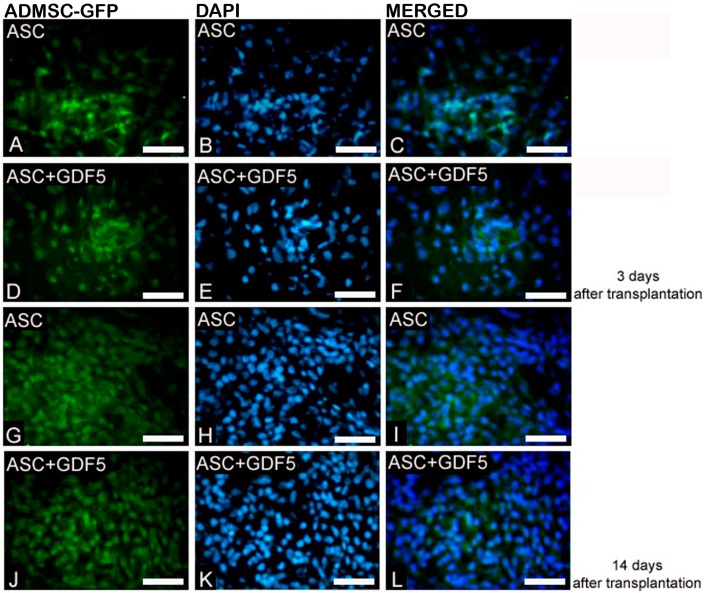
ADMSC-GFP Migration to TR on the 3rd and 14th days after injury: Observe the presence of ADMSC-GFP (**A**,**D**,**G**,**J**) in the ASC and ASC+GDF5 groups. Visible higher numbers of cells can be observed on the 14th day in both groups. DAPI: Nuclei marking (**B**,**E**,**H**,**K**). Merged images of ADMSC-GFP with nuclei marked with DAPI (**C**,**F**,**I**,**L**). Bar = 50 μm.

In the present study, significant alterations in the ECM of the Achilles tendon were observed after 14 days since the partial transection, both from the application of the ADMSC isolated in the injured region and from the application of the ADMSC associated with GDF-5. The cell migration assay demonstrated the presence of ADMSC-GFP in tendon sections of both groups, ASC and ASC+GDF5, on the 3rd and 14th days after injury (Figure 3).

#### 3.2.2. Real-Time PCR Array

**Figure 4:**
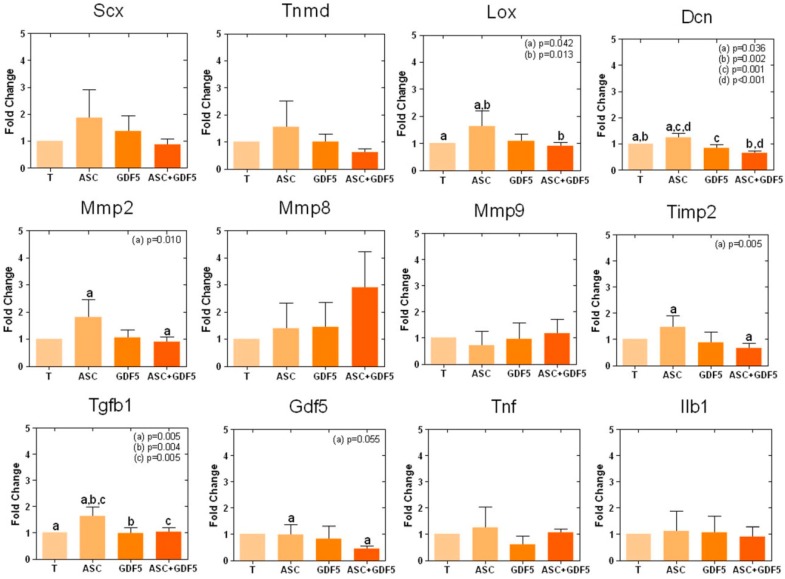
RT-PCR array for expression analysis of 12 genes in transected tendons: 50% of the genes analyzed were altered. The ADMSC increased expression of *Lox*, *Dcn*, and *Tgfb1* when compared to the other groups. Compared only to the ASC+GDF5 group, ADMSC increased the expression of *Mmp2*, *Timp2*, and *Gdf5*. The same letter between the groups corresponds to a significant difference between them.

The 12 genes expression analysis, obtained through the RT-PCR array (Figure 4), showed that the ADMSC application up-regulated the expression of the *Lox*, *Dcn*, and *Tgfb1* genes compared to the T group, and of the *Mmp2*, *Timp2*, and *Gdf5* genes in relation to the ASC+GDF5 group.

#### 3.2.3. Dosage of Hydroxyproline

**Figure 5:**
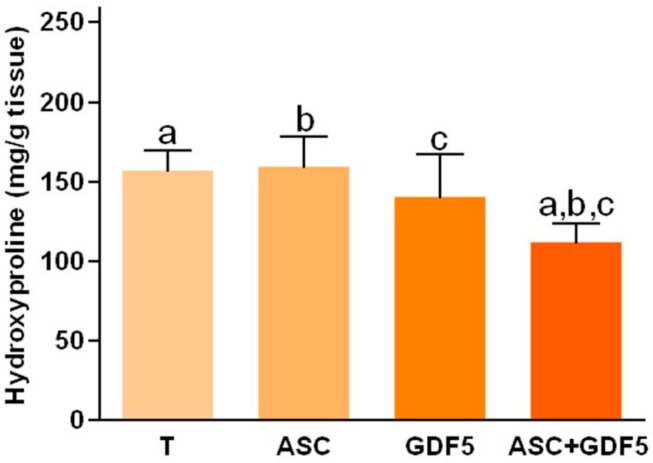
Tendons hydroxyproline concentration: Observe the lower value in the ASC+GDF5 group in relation to the other groups. The same letter among groups corresponds to a significant difference between them (*p* ≤ 0.05).

Regarding the hydroxyproline dosage (Figure 5), which infers the concentration of total tissue collagen (mg/g of tissue), a lower value was demonstrated in the ASC+GDF5 group in relation to all other groups.

#### 3.2.4. Birefringence Measurements

**Figure 6:**
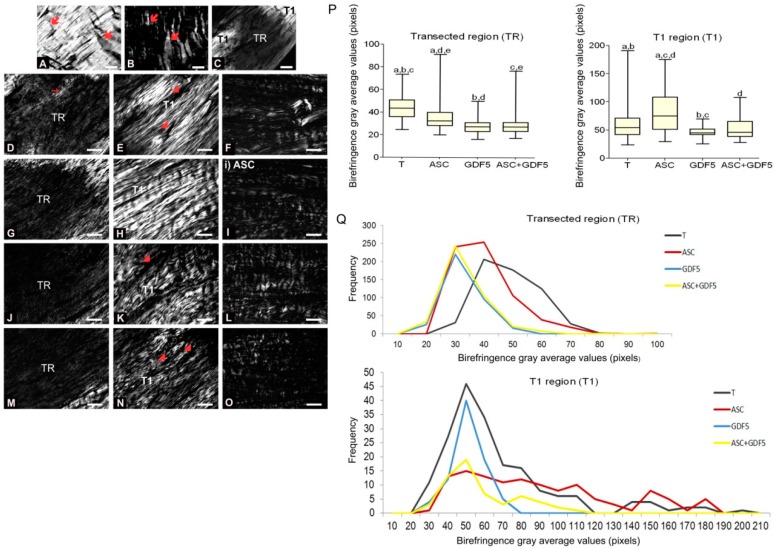
Images of tendons using polarization microscopy. (**A**) group N: Birefringence of the collagen fibers in the proximal region of the calanear tendon. The variation in gray levels is due to the crimp and the degree of aggregation of the collagen fibers (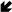); observe the crimp (**B**) by positioning the largest axis of the tendon parallel to one of the polarizers: The same region observed in (**A**). (**C**) Panoramic image of the transected tendon for identification of the transection region (TR) and the proximal and distal transition region (T1). Groups T (**D**–**F**), ASC (**G**–**I**), GDF5 (**J**–**L**), and ASC+GDF5 (**M**–**O**). Observe the complete disorganization of collagen fibers in TR. The TR from different groups (**D**,**G**,**J**,**M**): Observe freshly formed collagen fibrils and an overlapping (↘) of this region with the thicker fibers present in T1. **T1** (**E**,**H**,**K**,**N**): Collagen fibers with a greater organization in relation to TR, however, with fragmentation presence (◢) mainly in groups T (**E**), GDF5 (K), and ASC+GDF5 (N). *Crimp* (**F**,**I**,**L**,**O**) from the collagen fibers observed on T1: Observe similar undulation patterns of the collagen fibers between the groups, represented by light and dark regions. The largest axis of the tendon was positioned at 45° in relation to the crossed polarizers as parallel to one of the polarizers (**B**,**F**,**I**,**L**,**O**). (**P**) TR birefringence measurements in T1: Same letter represents significant differences between groups (*p* ≤ 0.05). (**Q**) Histogram of the frequency and birefringence values showing differences in the distribution of values in the different groups. Bars = 100 μm and 200 μm (a).

In the birefringence measurements obtained through polarization microscopy (Figure 6), differences were observed in the collagen fiber organization of the tendons of the different groups. The tendons analyzed areas were characterized as follows: Transection region (TR), where partial transection of the collagen bundles was performed; and proximal and distal transition region (T1), which are located in the adjacency of the TR. Considering the TR, the T group presented higher birefringence values (gray average values in pixels) in relation to the other transected groups. The ASC group, in relation to the groups, GDF5 and ASC+GDF5, had a higher value of birefringence. Regarding the organization of the collagen bundles in T1, the ASC group presented greater birefringence in relation to the other groups, and GDF5 presented a lower value also in relation to the T group. Regarding the organization pattern of the crimp, no significant difference was observed between groups.

#### 3.2.5. Linear Dichroism Measurements

**Figure 7:**
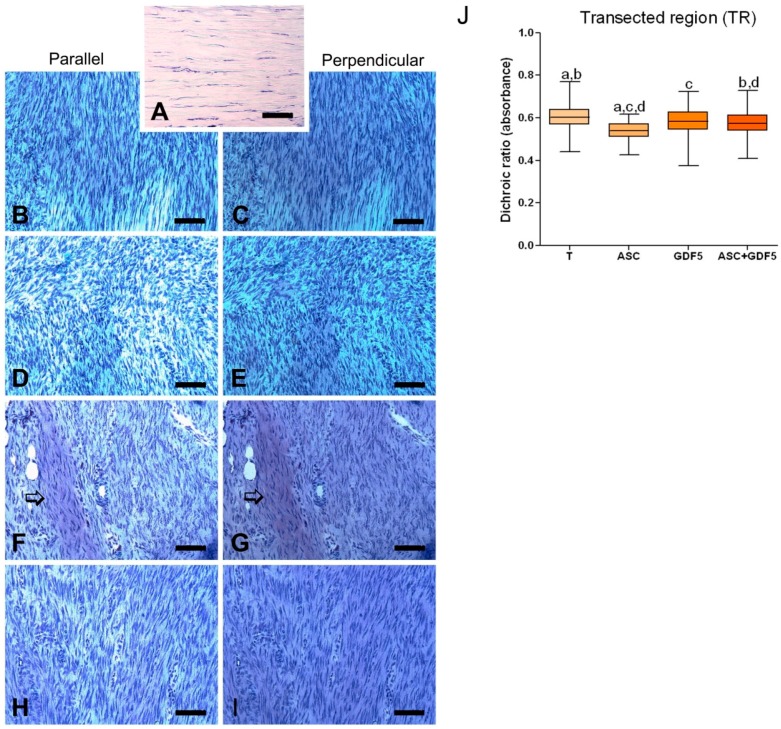
Linear dichroism (DL) of AT-stained tendon sections and analyzed under polarization microscopy. The largest axis of the tendon was placed in the parallel (**B**,**D**,**F**,**H**) and perpendicular (**C**,**E**,**G**,**I**) position relative to the polarized light plane. Observed DL is typically more intense in the cuts in the perpendicular position. Group N (**A**) is observed under common light microscopy due to the low amount of PG. (**J**) Dichroic index (ID) calculated through linear dichroism measurements (absorbance) performed on tendons TR: Greatest value observed in group T. Same letter between groups corresponds to a significant difference between them (*p* ≤ 0.05). Bar = 100 μm.

The dichroic ratio (DR) calculated from the linear dichroism measurements performed in sections stained with toluidine blue (Figure 7), through the use of polarization microscopy, showed differences in the organization of the PG present in the TR of the tendons. The T group presented higher DR when compared to the ASC and ASC+GDF5 groups, followed by the GDF5 group, with a higher value in relation to the ASC group.

#### 3.2.6. CatWalk System

**Figure 8:**
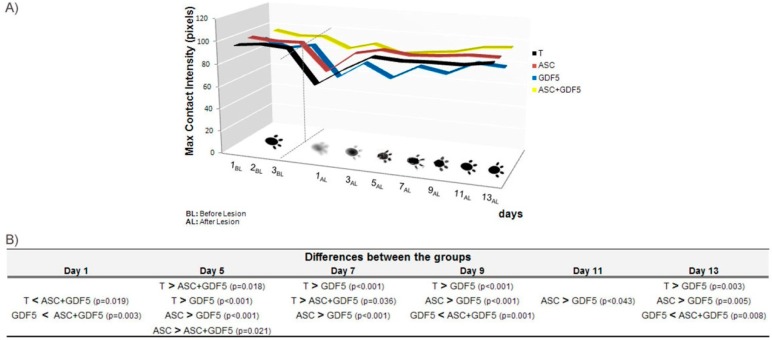
(**A**) Maximum contact intensity of the paw of the animals during walking, obtained through the CatWalk system. The measurements were taken three days before the injury to obtain the normal walk pattern, and on the 1st, 3rd, 5th, 7th, 9th, 11th, and 13th days after the tendon transection. Observe the marked decrease in contact pressure of the animals’ paw on the day after surgery, with a higher value of the ASC+GDF5 group compared to the T group. Except for the GDF5 group, observe the complete recovery of the normal walk pattern of the animals in the other groups on the 13th day. (**B**) Comparisons between groups with significant differences observed between the 1st and 13th days.

In the functional analysis obtained through CatWalk (Figure 8), differences were observed in the rats’ gait in the different groups. Considering the maximum contact intensity of the paw (pixels) during gait, the ASC+GDF5 group presented a higher value in relation to the T and GDF5 groups 24 h after the transection. Between the 5th and 13th days after transection, the ASC group presented higher values in relation to the GDF-5 treated groups, although without a significant difference in relation to the T group.

#### 3.2.7. Biomechanical Parameters

**Figure 9:**
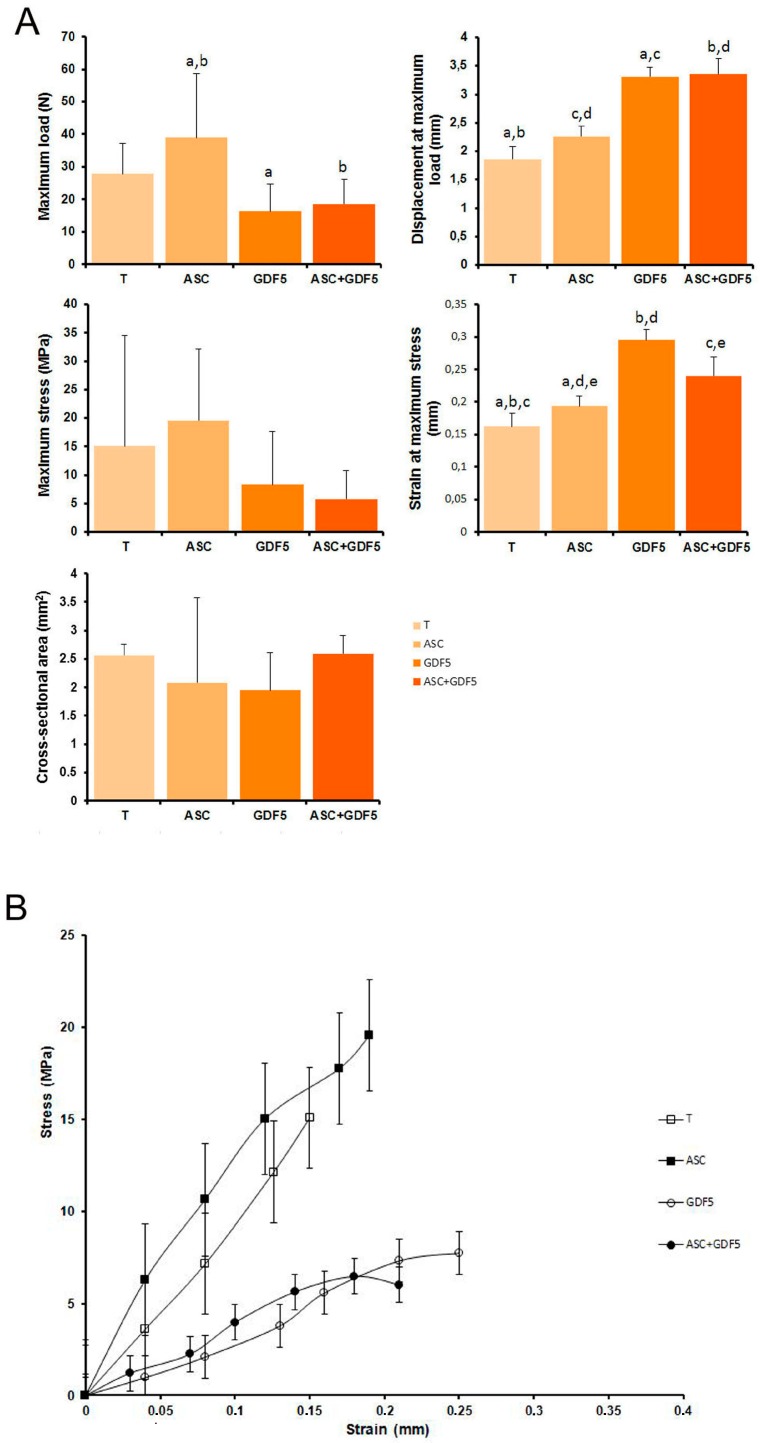
(**A**) Biomechanical properties of the tendons: Significant differences can be observed between the groups for the parameters of maximum load, displacement, and strain. The same letter between the groups corresponds to a significant difference between them (*p* ≤ 0.001). (**B**) Stress-strain curve: Tendons treated with ADMSC presented lower deformation at higher stress in comparison to the other groups. Standard deviations are represented by vertical bars.

The biomechanical analyses of the Achilles tendon (Figure 9A) showed significant differences between the groups considering some parameters. Regarding the maximum rupture load, the ASC group presented a higher value in relation to the GDF5 and ASC+GDF5 groups. Considering the displacement and strain parameters, the groups treated with GDF-5 presented higher values in relation to the T and ASC groups. No difference was observed in the cross-sectional area between the groups. In the stress-strain curve (Figure 9B), tendons treated with ADMSC presented lower deformation at higher stress in comparison to the other groups.

## 4. Discussion

Several current therapeutic techniques have been applied with the objective of improving tendon healing with partially satisfactory results considering its functional repair. The healed tendon is often characterized by functional impairment and a risk of re-rupture, mainly at the site of injury or near the injury region [13]. Recently, ADMSC have been proposed as a new treatment to improve this repair process due to its multipotency, cultivation facility, high yield, and, because they originate from adult donors, lack of ethical issues. In the present study, in the characterization of the cells before application, ADMSC expressed the positive surface markers, CD90 and CD105, and low expression of the negative marker, CD45. ADMSC also differentiated in chondrocytes, osteoblasts, and adipocytes, showing multilineage differentiation. Nucleoli were also observed in the ADMSC, indicating their synthesis activity, as an interesting profile of genes expression was exhibited by the ADMSC in vitro due to the expression of *Lox*, *Dcn*, *Mmp2*, *Timp2*, and *Tgfb1* genes, and no expression of the genes, *Scx*, *Tnmd*, *Mmp9*, *Gdf5*, *Tnf*, and *Ilb1*.

The transplanted ADMSC-GFP migrated to the transected tendon region in response to the specific microenvironment of the injury, characterized by the intense initial inflammatory process [44,45], and remained until the 14th day of the repair process. Thus, the alterations found in the groups after ADMSC transplantation are directly associated with the incorporation of these cells into the tendon. It is important to mention that at the time of tendons application, a mean of 80% of viable ADMSC were transplanted. According to Gimble et al. [18], the use of ADMSC is promising in several repair processes because of their potential for differentiation in different cell lines, as well as the secretion of growth factors and other signaling molecules.

In the present study, ADMSC increased the organization of collagen fibers in the injury adjacent region, which was reflected in the biomechanical properties of the tendons. Compared to the control transected tendon, tendons treated with ADMSC were apparently more resistance to traction, with lower deformation at higher stress. The organization and mechanical properties of the tendon are determined mainly by the orientation of fibrils and collagen fibers, fiber diameter, number of intra- and inter-molecular crosslinks that guarantee high tensile strength, total collagen content, the amount of PG, and the presence of other non-collagen proteins, such as cytokines, growth factors, and structural proteins [8,9,46,47,48]. Some studies have shown the effectiveness of ADMSC in some models of tendon injury. Results from Del Bue et al. [49] showed the benefits of ADMSC application, in association with a platelet concentrate, in the functional recovery of horses’ tendons. In a model of rabbits’ calcaneal tendon injuries, the application of ADMSC associated with a gel composed of a plasma rich in platelets increased the biomechanical resistance of the tendons in the 4th week of the healing process and increased the amount of collagen type I, VEGF, and FGF [7]. Uysal and Mizuno [50] also showed the effectiveness of the application of ADMSC in isolation at the site of the tendon injury, in which there was an increase in tendon resistance to tension, increase of angiogenic growth factors, and differentiation of stem cells into tenocytes and endothelial cells.

The application of ADMSC in association to GDF-5 led to a lower concentration of total collagen and a low degree of organization of ECM tendon, evidenced by the low birefringence of the collagen fibers when compared to the group that received only the ADMSC. Our results indicate that the application of GDF-5 alone and when associated with ADMSC both reduce tissue organization, resulting in inferior biomechanical properties when compared to control transected tendons, as well as in relation to the tendons transected and the ADMSC treated ones. However, several studies have demonstrated the modulatory action of GDF-5 on the tendon healing process due to its effect on cell migration and adhesion, differentiation, proliferation, and angiogenesis [28,29,31,51].

The beneficial role of GDF-5 was also demonstrated in the study by Mikic et al. [52] using deficient GDF-5 mutant mice, in which the tendons of these animals showed 40% less collagen compared to control tendons, as well as biomechanically less resistant tendons. A study by Chhabra et al. [31] reported that deficient GDF-5 mice presented until the 8th week of the healing process of the calcaneal tendon, a delay of one to two weeks in this process, characterized by a lower collagen concentration and smaller fibrils diameter collagen, lower amount of glycosaminoglycans (GAG), lower cell and blood vessel density, greater amount of fat, and lower biomechanical resistance until the 12th week. Rickert et al. [53] used gene therapy for the endogenous production of GDF-5 by genetically modified cells, which were applied to the injured tendon. Although the tissue quality was slightly greater than the control tendon, the presence of cartilaginous tissue was observed in the 8th week, disappearing in the 12th week of the healing process. Thus, it is possible to conclude that the lower biomechanical properties observed in the GDF-5 treated groups are directly related to the smaller organization of the fibers [48], as well as to the lowest total collagen content.

A lower dichroic ratio was observed in the ADMSC treated groups compared to the only transected group, indicating changes in the GAG arrangement in the collagen fibers present in the tendons of these groups. GAG chains are constituents of PG, which may be associated with collagen fibers through non-covalent interactions. Additionally, in normal tendons, the GAG chains present in PG are predominantly parallel to the major axis of the tendon [3]. A study by Mello and Vidal 40] showed differences in the arrangement of GAG up to 110 days after calcaneal tendon injury in rats. Thus, the lower dichroic ratio observed in the group treated with ADMSC alone suggests that not all GAG chains are neatly parallel to the major axis of the tendon, corroborating to the smaller organization of newly formed collagen fibers in the TR of the same group. The small PG play an important role in the fibrilogenesis of the collagen, regulating the growth in the fiber diameter [6]. It is important to emphasize that both groups treated with GDF-5 presented a higher dichroic ratio in relation to the ASC group, although less organization of the collagen fibers was observed in these groups treated with GDF-5.

Growth factors are small peptides, which generally transmit signals between cells, and are thus molecules that modulate cellular activity. In general, growth factors can regulate cell activity through several mechanisms, such as mitogenic activity, cell differentiation, cell migration, and gene regulation; and also play an important role in cellular chemotaxis and ECM synthesis [54,55]. The application of growth factors with the goal of improving the tendon healing process is still experimental, and has been restrictive to in vitro experiments and animal models [55,56]. The association of GDF-5 to the ADMSC used in this study was mainly based on the in vitro results of Park et al. [19]. An increase in ADMSC proliferation after GDF-5 application, and an increase in the amount of type I collagen, decorin, and aggrecan was seen, as well as markers of tendon cells, such as Scx, Tnmd, and tenascin-C, which have indicated differentiation of ADMSC in tenocytes [19]. However, in vivo, our results demonstrated low effectiveness of both the application of GDF-5 alone and after its application in association with ADMSC.

The literature points to a limitation in the application form and frequency of growth factors during tendon healing due to the relatively short half-life of these factors, which would only allow the short-term modulation of their biological effects [53]. Results from Forslund et al. [30] showed interesting data after a single injection (six h after the tendon injury) of different doses (0.4, 2, and 10 μg) of GDF-5, -6, and -7. On the 8th day after surgery, all factors increased the biomechanical resistance of the rats’ calcaneal tendon after the application of the higher doses of 2 and 10 μg, in addition to observing a decrease in the inflammatory process. Yet, after the application of 10 μg of the factors in this same study, bone and cartilaginous formation in the tendons was observed. In the present study, 500 ng of GDF-5 was used based on the data of Park et al. [19] that treated the culture of ADMSC with different concentrations of GDF-5 (0–1000 ng/mL).

The application of GDF-5 in this study occurred 24 hours after tendon transection. Considering the important participation of the GDF-5 in tendon repair, mentioned above in several studies, and as most of our results indicate a greater effectiveness of applying only ADMSC during this phase of the tendon repair process, important variables should be GDF-5: The animal model used, the model of tendon injury, including the extent of the injured area, the origin of the growth factor, and the dosage and form of administration of the same, as well as the period elapsed between injury and the application of the growth factor [57]. According to some authors, the administration of the growth factor after one week of surgery results in an improvement in the quality of the healing process, including improvement in the biomechanical properties of the tendon and the structural organization of its ECM [58,59,60,61].

The most striking result in the functional analysis obtained through CatWalk showed a greater intensity of the rats’ paws’ contact pressure during gait 24 h after transection, possibly due to the decrease of pain after treatment with the ADMSC associated with GDF-5 in comparison to the control group. Treatment based on this association may have contributed to the reduction of acute inflammation and post-surgical edema, as well as decreased pain due to the reduction in the concentration of prostaglandins E2, nitric oxide activity, and reduction of free radicals [62]. These previously mentioned factors, although speculative, corroborate with the data of Forslund et al. [30], which showed that a single injection of GDF-5 during tendon repair decreased the inflammatory process, and with the data of Schneider et al. [17] that described immunomodulatory effects of the ADMSC.

The expression of 12 different genes related to inflammation, the remodeling of ECM, and the differentiation of ADMSC into tenocytes were studied to elucidate some molecular mechanisms involved in the repair of tendons. Our results demonstrated that ADMSC up-regulated the *Dcn* gene expression in comparison to other groups. The *Dcn* is a PG with a well-established structural function as it mediates the lateral fusion of collagen fibrils, contributing to the formation of mature collagen fiber with a larger diameter and, therefore, greater biomechanical resistance [63]. The lower expression of *Dcn* observed in the tendons treated with the ADMSC in association with GDF-5 may be directly related to the lower organization of the collagen fibers, resulting in lower biomechanical properties when compared to the control transected tendons, in relation to the ADMSC treated tendons. The application of ADMSC in association with GDF-5 also down-regulated the *Lox* gene expression compared to the ASC group, corroborating to the lower organization of collagen fibers in the ASC+GDF5 group. Herchenhan et al. [64] have demonstrated the direct role of *Lox* in collagen fibrilogenesis, showing that the inhibition of this enzyme activity harms the formation of the characteristic pattern of fibrils, leading to a decrease in the biomechanical resistance of tendon-like tissues constructed through bioengineering. It is worth mentioning that the *Lox* expression was higher after the treatment with ADMSC in comparison to the control transected tendon. It seems clear that there is a relation of the lower *Gdf5* gene expression in the ASC+GDF5 group with the lower ECM organization and biomechanical parameters observed when compared to the ASC group [28,29,30,31].

Regarding the expression of genes related to tissue remodeling, *Mmp2*, *Mmp8*, *Mmp9*, and *Timp2*, higher expression of *Mmp2* and *Timp2* was observed in the ASC group in relation only to the ASC+GDF5 group. The literature describes the increased expression and activity of these MMPs during tendon repair processes [8], since previous studies of our group showed a direct relationship between the greater activity of MMP-2 and greater organization of the ECM of tendons [36,65,66], corroborating with the results from the present study. It is important to emphasize that the larger organization of the collagen fibers in the T1 region was also observed in the group treated with ADMSC only.

The application of ADMSC also up-regulated the *Tgfb1* gene expression, another important gene whose expression modulates important processes during tendon repair. *Tfb1* acts on the regulation of cell proliferation, differentiation, and migration, apoptosis, GAG deposition, and stimulates the production of collagen by fibroblasts [67,68,69,70], influencing the tissue healing cascade [71]. *Tgfb1* is also involved in increased expression of tissue inhibitors of MMP (*Timp*) and *Lox* in different cell types, including in vitro fibroblasts [72], corroborating our results that showed a relation between the higher expression of *Tgfb1*, and increased expression of *Lox* and *Timp2* after ADMSC application.

We observed a strong trend towards higher expression of the *Scx* and *Tnmd* genes in tendons after ADMSC application in relation to the control group, supposing differentiation of ADMSC applied and/or endogenous stem cells into tenocytes. ADMSC have proven to modulate the host’s “stem cell niche” by stimulating and recruiting endogenous stem cells to the injured site, promoting their differentiation [18]. *Scx* has recently been reported as a marker of progenitor cell populations in tendons, being a transcription factor present in tendons from development to adulthood [73]. *Scx* is a key regulator in the differentiation of tenocytes, whose expression is highly induced through the signaling pathway involving *Tgfb1* [74], which was increased in the ASC group. According to Shukunami et al. [75], *Scx* positively regulates the expression of *Tnmd*, which is a transmembrane protein specifically expressed in dense connective tissues, such as tendons, ligaments, epimysium of skeletal muscle, cornea, and sclera [76,77,78,79,80], indicating the differentiation of ADMSC into tenocytes.

Our data showed the influence of the application of ADMSC isolated and in association with GDF-5 in the expression of 50% of the analyzed genes, *Dcn*, *Gdf5*, *Lox*, *Tgfb1*, *Mmp2*, and *Timp2*. ADMSC in vitro presented, with exception of the *Gdf5* gene, a similar gene expression profile of those five genes in comparison to the *Scx*, *Tnmd*, *Mmp9*, *Tnf*, and *Ilb1* genes. The literature describes that the ADMSC behavior seems to be modulated by the presence of molecules at the site where they are injected, such as cytokines, chemokines, peptides, and, mainly, growth factors [67,68]. Other studies also demonstrate the paracrine and immunomodulatory effects of ADMSC at the site of injury to promote tendon regeneration [16,17].

In conclusion, the application of ADMSC up-regulated the *Dcn*, *Lox*, and *Tgfb1* genes expression, which contributed to a higher collagen fiber organization and tendon biomechanics. The association of ADMSC with GDF-5 down-regulated the *Dcn*, *Gdf5*, *Lox*, *Tgfb1*, *Mmp2*, and *Timp2*, which contributed to an improvement of the rats’ gait 24 h after the injury and impaired the organization and biomechanics of tendons. Although the literature describes the benefic effect of GDF-5 for the tendon healing process, our results show that its application, isolated or associated with ADMSC, cannot improve the repair process of partial transected tendons, indicating the higher effectiveness of the application of ADMSC in injured Achilles tendons.
